# Meeting the demand of the future: a curriculum to stimulate interest in careers in primary care internal medicine

**DOI:** 10.1080/10872981.2017.1340780

**Published:** 2017-07-03

**Authors:** Mary R. Hawthorne, An Dinh

**Affiliations:** ^a^ Internal Medicine Clerkship, University of Massachusetts Medical School, Worcester, MA, USA; ^b^ Institutional Analyst III, University of Massachusetts Medical School, Worcester, MA, USA

**Keywords:** Primary care, medical student, career choice, curriculum, medical education

## Abstract

**Background**: There is a growing need for primary care physicians, but only a small percentage of graduating medical students enter careers in primary care.

**Purpose**: To assess whether a Primary Care Intraclerkship within the Medicine clerkship can significantly improve students’ attitudes by analyzing scores on pre- and post-tests.

**Methods**: Students on the Medicine clerkship at the University of Massachusetts Medical School participated in full-day ‘intraclerkships’,to demonstrate the importance of primary care and the management of chronic illness in various primary care settings. Pre-and post-tests containing students’ self-reported, five-point Likert agreement scale evaluations to 26 items (measuring perceptions about the roles of primary care physicians in patient care and treatment) were collected before and after each session. Eleven intraclerkships with 383 students were held between June 2010 and June 2013. Responses were analyzed using the GLM Model Estimate.

**Results**: Results from the survey analysis showed significantly more positive attitudes toward primary care in the post-tests compared to the pre-tests. Students who were satisfied with their primary care physicians were significantly more likely to show an improvement in post-test attitudes toward primary care in the areas of physicians improving the quality of patient care, making a difference in overall patient health, finding primary care as an intellectually challenging field, and in needing to collaborate with specialists. Older students were more likely than younger students to show more favorable answers on questions concerning the relative value of primary care vs. specialty care.

**Conclusions**: A curriculum in Primary Care Internal Medicine can provide a framework to positively influence students’ attitudes toward the importance of primary care, and potentially to influence career decisions to enter careers in Primary Care Internal Medicine. Ensuring that medical students receive excellent primary care for themselves can also positively influence attitudes toward primary care.

## Introduction

There is little doubt that there is a growing need for primary care physicians. The population is aging, and patients previously un-insured have been re-entering the healthcare system under the US Affordable Care Act [1–3]. However, fewer than 20% of American medical school graduates indicated that they would choose careers in primary care [4–7]. The reasons for this are many. The median debt for graduating medical students is $160 000 and more than a third of students owe more than $200 000 [8]. Up to now, specialists have earned significantly more than primary care physicians [9].. The median age for primary care physicians is increasing, and an insufficient number of new physicians are entering the workforce to replace older physicians as they reach retirement age [10]. In addition, the need for increased documentation, implementation of practice guidelines, and increased physician time spent on clerical duties with electronic medical records has resulted in each primary care physician needing to reduce the number of patients seen [11]. Yet, studies have shown that access to primary care saves a tremendous amount of money for the healthcare system through improved screening for preventable illness, lower rates of Emergency Department use, lower rates of avoidable hospitalizations, and mitigation of the effects of lower socioeconomic status on illness [12–14]. Medical school class sizes are increasing, but Medicare has not increased funding for residencies, nor has there been a requirement to place emphasis on primary care training [15]. Students often view careers in primary care as less prestigious than specialty careers.

In its 2010 annual national survey of clerkship directors, the Clerkship Directors in Internal Medicine found that 85% of responding schools offered ambulatory education to students in either the third or fourth year of medical school, or 72% of those required ambulatory training as part of the core Internal Medicine clerkship. Hauer et al. found that a third of students who had an outpatient Internal Medicine clerkship felt that it made a career in General Internal Medicine *less attractive* [16]. Students spending time in General Internal Medicine offices may see physicians who struggle to keep up with patient care and documentation, and may see specialty careers as more attractive. Clearly, just placing students in ambulatory offices for primary care training and hoping for the best in terms of recruitment to careers in primary care isn’t an effective strategy on its own.

This study sought to assess whether adding a full-day Primary Care Internal Medicine curriculum to time spent on ambulatory Medicine in the third year of medical school can improve students’ attitudes toward primary care.

## Methods

Students at the University of Massachusetts Medical School experience their Medicine clerkship as part of a 15-week integrated clerkship, combined with Neurology. Students spend eight weeks on Inpatient Medicine, two weeks in Primary Care Ambulatory Medicine, three weeks on Neurology, and two weeks in electives in a thematic section called Care of Adults. Students on the rotation return to the University for a full-day curriculum in Primary Care. The curriculum includes sessions on the importance of primary care in population health; the management of chronic illness (diabetes, heart failure, and chronic obstructive pulmonary disease) in various practice settings (University-based practice, community-based office practice, or private practice); the use of motivational interviewing to help patients with management of obesity; and the role of primary care physicians as patient advocate when there is a difference of opinion between the primary care physician and specialist. The format for the sessions was a combination of interactive lecture and small group discussions, and the sessions were facilitated by primary care physicians selected for being positive role models. In addition to pre- and post-intraclerkship surveys detailed below, the intraclerkship includes a discussion with students about what qualities they think define excellent primary care, and their definitions are compared with the World Health Organization prescribed ideals of primary care to provide accessibility, coordination of care, longitudinality and comprehensiveness of care.

## Measures

Data for these analyses were extracted from students’ self-reported, five-point Likert agreement scale (‘strongly disagree’ (1) to ‘strongly agree’ (5)) evaluations collected before and after each session. Course leaders and content experts selected twenty-six items seeking students’ opinions about their perceptions of the roles of primary care and primary care physicians in patient care and treatment. One item was later excluded after being deemed not relevant to the curriculum. Eight items were on a reversed scale and were later converted to reflect the higher score being coordinated with a better perception. The evaluation forms were completed using E*Value, an automated, confidential online program.

Demographic information such as gender, age, and race/ethnicity was subsequently obtained from our student enrollment system of records. Age was analyzed as a continuous variable as well as a bimodal categorization to differentiate between those students who enter graduate study essentially immediately after college graduation (less than 26 years old), compared to those who spend time before medical school pursuing other activities (26 years and older). All non-white students were collapsed into one group, given the small subsample sizes. Other experiences such as having a primary care doctor, the level of satisfaction with one’s own primary care doctor, having a primary care physician as a family member, and prior exposure to a Family Medicine clerkship were included as risk factors in the analyses.

## Statistical analysis

Descriptive analysis included frequencies and prevalence of demographic factors. Instrument validity was assessed initially using face validity, where items were created, discussed, and selected by clerkship directors, instructors, and other content experts, based on their expertise and experiences. At the item level, non-parametric Wilcoxon signed rank tests were performed to evaluate any change in rating before and after clerkships. Factorial analysis was conducted and factors were determined based on an eigenvalue of greater than 0.25 and on the scree test, subjected to a VARIMAX orthogonal rotation. Two factors were identified: (1) advocacy for the role of primary care, and (2) the importance of primary care compared with other specialties. Factors’ validity was later assessed using Cronbach reliability and item-to-group correlations. A composite mean of rating scores for each factor was calculated. A mixed-effect generalized linear model was used to assess the change of the ratings post- vs. pre-test and the impact of demographic and other risk factors on the rating score. Statistical significance levels were set at a = 0.05. All analyses were performed using SPSS 22.0, IBM Inc.

## Results

Our students in this survey included more who were female (54.0%), white (76.2%), or had primary care physicians (71.4%), with the average age at the event of 23.4. A total of 71% of students had a primary care doctor, and 53.1% were satisfied or very satisfied with their PCP. A total of 37.2% reported to have a physician and 28.2% to have a PCP in the family. A total of 34% had taken their Family Medicine clerkship before this event (Table 1).

**Table 1.** Demographic and other baseline characteristics.

Demographic and other baseline characteristics	
Gender, % (n)	
Female	54.0% (207)
Male	46.0% (176)
Race, % (n)	
White	76.2% (291)
Non-white	23.8% (91)
Age at event, mean (sd)	23.4 (3.38)
26 years and older, % (n)	52.7% (196)
<26 years old, % (n)	47.3% (176)
Have a primary care physician? % (n)	
Yes	71.4% (274)
No	28.6% (105)
Are there any physicians in the family? %(n)	
Yes	37.2% (145)
No	61.8% (235)
Are there any primary care physicians in the family? % (n)	
Yes	28.2% (107)
No	71.8% (272)
Are you satisfied with the care received from your primary care physician? % (n)	
Yes	53.1% (234)
No	46.9% (137)
Prior exposure to Family Medicine clerkship	
Yes	34.1% (125)
No	65.9% (242)

Of all students attending the intra clerkship, two students completed the pre- but not the post-survey, two students did not complete the pre- but completed the post- surveys, and seven students completed neither pre- nor post- survey, leaving us a very high response rate of 97.1%, which reduced any potential selection bias.

Table 2 provides student ratings at the item level. Students started with ratings on the positive side (mean from 3.17 to 4.28 on a scale of 5) except in primary care as a career choice (between 2 and 3). The students’ ratings further increased statistically significantly post vs. pre, in the majority of items, including that the primary care physician provides the first entry to the health care system (p < .0001), dedicates a significant amount of time to improving the quality of patient care (p < .0001), makes a significant difference in patient health while doing preventive care (p < .0001), does not play a lesser role than subspecialists in managing health issues (p < .0001), takes care of complex medical problems (p < .0001), should propose opinions about the management of complicated medical patients (p < .0001), and should intervene and stay involved after patients receive care from other physicians or subspecialists (p < .0001). After the event, students also increased their ratings of their recognition of the roles and challenges of primary care, the high workload, sometimes difficult schedule, and future importance (p < .0001).

**Table 2.** Item analysis*.

		Pre	Post	Z	p
1	Patients usually first enter the health care system through primary care	3.30	3.63	7.03	<.0001^†^
2	Primary care physicians dedicate a significant amount of time to improving the quality of patient care	3.90	4.24	7.90	<.0001^†^
3	Preventive health care by primary care physicians makes a significant difference in overall patient health	4.28	4.43	4.58	<.0001^†^
4	Primary care internists manage health problems of lesser importance than those managed by subspecialists^a^	3.88	4.11	4.72	<.0001^†^
5	Patients with complex medical problems are best taken care of by subspecialists^a^	3.17	3.40	4.99	<.0001^†^
6	When there is a difference of opinion regarding the management of a complicated medical patient, the primary care physician should always defer to the subspecialist’s opinion^a^	3.58	3.98	8.87	<.0001^†^
7	It is impossible to be competent in such a wide field as Primary Care Internal Medicine^a^	3.92	4.02	2.03	.04^†^
8	Primary Care Internal Medicine is not a very intellectually challenging or stimulating specialty^a^	4.05	4.08	.79	.43
9	Primary care internists often need to intervene in the care that patients receive from other physicians	3.22	3.29	1.45	.15
10	When a primary care physician needs to change a medication prescribed by a subspecialist, he/she should notify the other physician of the change	3.95	4.01	1.18	.24
11	Hospitalists should involve primary care physicians in medical decision-making in the hospital setting	3.68	3.86	4.21	<.0001^†^
12	Patients prefer that their primary care physicians remain involved in the care they receive from subspecialists	4.04	4.14	3.05	<.0001^†^
13	Patients who see a primary care physician are likely to be healthier than those who do not have a primary care physician	4.10	4.37	6.42	<.0001^†^
14	Scolding patients who do not take care of themselves helps motivate them to change their behaviors^a^	3.76	3.93	3.16	<.0001^†^
15	Primary care internists are poorly valued by the rest of the medical profession^a^	2.93	3.32	7.59	<.0001^†^
16	Medical secretaries and medical assistants make a large contribution to the care of patients	3.96	4.08	3.22	<.0001^†^
18	The primary care internist is clinically competent to provide most of the health care an individual may require	3.83	3.94	2.87	<.0001^†^
19	Primary care internists make decisions in highly uncertain circumstances	3.43	3.54	3.37	<.0001^†^
20	Chronic disease management by primary care physicians significantly affects outcomes	4.19	4.30	2.76	.01^†^
21	Primary care internists have little control over their schedules^a^	3.30	3.66	7.35	<.0001^†^
22	Primary care internists have a large work overload	3.88	3.57	6.83	<.0001^†^
23	The role of primary care in the future is likely to be more important	4.14	4.27	3.83	<.0001^†^
24	It is appropriate to have a Primary Care Intraclerkship within the Medicine Clerkship	3.85	3.90	1.74	.08
25	I would like to become a primary care physician in the future	2.94	2.92	.38	.70
26	Primary care internal medicine is my first career choice	2.37	2.46	2.83	<.0001^†^

*Scale 1–5: 1: Extremely unlikely, to 5: Extremely likely.

^a^Reversed coding items. These items’ scale was subsequently reversed as in the table in order to associate higher scales with better perception.

^†^Denotes statistically significantly different post vs. pre, at significant level α = 0.05.

Student ratings remained unchanged in some items, namely ‘Primary Care Internal Medicine is not a very intellectually challenging or stimulating specialty’ (p = .43), ‘Primary care internists often need to intervene in the care that patients receive from other physicians (p = .15), ‘When a primary care physician needs to change a medication prescribed by a subspecialist he/she should notify the other physician’ (p = .24), ‘It is appropriate to have a Primary Care Intraclerkship within the Medicine Clerkship’ (p = .08) and ‘I would like to become a primary care physician in the future’(p = .70).

Factor analyses identified two areas into which student responses sorted themselves. Factor 1 consisted of 15 items (λ = 4.8, Cronbach α = 0.85; item to group correlation run from 0.26 to 0.70) reflecting students’ opinions on the advocacy role of primary care. These were items such as ‘Primary care internists often need to intervene in the care that patients receive from other physicians’, ‘Hospitalists should involve primary care physicians in medical decision-making in the hospital setting’, ‘Patients prefer that their primary care physicians remain involved in the care they receive from subspecialists’ and ‘Chronic disease management by primary care physicians significantly affects outcomes.’ A session in the curriculum features small group discussions of cases in which a primary care physician had a difference of opinion from a subspecialist, and needed to decide whether to speak up to intervene in the patient’s care. Factor 2 accounted for nine items exhibiting students’ perceptions of the importance of primary care medicine compared to other specialties (λ = 2.4, Cronbach α = 0.71, item to group correlation running from 0.26 to 0.56). These items are detailed in Table 3. Composite ratings for both areas (those which evaluated the advocacy role of primary care and those which evaluated the importance of primary care compared with other specialties) increased significantly after the event, as compared with pre-tests (3.89 compared to 3.77 and 3.79 compared to 3.54, p values <.0001, respectively), (Table 4).

**Table 3.** Factoral analysis.

		Factor 1	Factor 2
1	Patients usually first enter the health care system through primary care		.28
2	Primary care physicians dedicate a significant amount of time to improving the quality of patient care	.42	
3	Preventive health care by primary care physicians makes a significant difference in overall patient health	.63	
4	Primary care internists manage health problems of lesser importance than those managed by subspecialists^R^		.44
5	Patients with complex medical problems are best taken care of by subspecialists^R^		.56
6	When there is a difference of opinion regarding the management of a complicated medical patient, the primary care physician should always defer to the subspecialist’s opinion^R^		.41
7	It is impossible to be competent in such a wide field as primary care internal medicine^R^		.40
8	Primary care internal medicine is not a very intellectually challenging or stimulating specialty^R^		.41
9	Primary care internists often need to intervene in the care that patients receive from other physicians	.26	
10	When a primary care physician needs to change a medication prescribed by a subspecialist, he/she should notify the other physician of the change	.27	
11	Hospitalists should involve primary care physicians in medical decision making in the hospital setting	.42	
12	Patients prefer that their primary care physicians remain involved in the care they receive from subspecialists	.66	
13	Patients who see a primary care physician are likely to be healthier than those who do not have a primary care physician	.56	
14	Scolding patients who do not take care of themselves helps motivate them to change their behaviors^R^		.26
15	Primary care internists are poorly valued by the rest of the medical profession^R^		.33
16	Medical secretaries and medical assistants make a large contribution to the care of patients	.46	
18	The primary care internist is clinically competent to provide most of the health care an individual may require	.48	
19	Primary care internists make decisions in highly uncertain circumstances		
20	Chronic disease management by primary care physicians significantly affects outcomes	.69	
21	Primary care internists have little control over their schedules^R^		.33
22	Primary care internists have a large work overload	.40	
23	The role of primary care in the future is likely to be more important	.68	
24	It is appropriate to have a Primary Care Intraclerkship within the Medicine Clerkship	.54	
25	I would like to become a primary care physician in the future	.45	
26	Primary Care Internal Medicine is my first career choice	.25

**Table 4.** Composite ratings of factors.

Factor	Pre-score (mean, sd)	Post-score (mean, sd)	P value
Factor 1 (Advocacy for the role of primary care)	3.77 (.027)	3.89 (.028)	<.0001
Factor 2 (Importance of primary care, compared to other specialties)	3.54 (.021)	3.79 (.024)	<.0001

Student opinions about the advocacy role of primary care appear to be higher in non-white students (p = .041), students older than age 26 (p = .048), and in students who have satisfactory experiences with their own primary care physicians (p < .0001), (Table 5).

**Table 5.** Factors affecting perception of the advocacy role of primary care physicians.

Parameter	Estimate	Std. Error	P value.	95%CI
Intercept	3.61	.039	.000	3.54–3.69
Post (vs. Pre)	.12	.034	.001	.05–.18
Age at event ≥26 (vs. younger than 26)	.07	.034	.048	.0006–.136
Non-white (vs. White)	.10	.041	.014	.02–.18
Has a satisfactory experience with PCP (vs. non-satisfactory)	.14	.036	.000	.073–.21

Student perceptions about the importance of primary care compared to other specialties seem to be higher in students older than age 26 (Table 6).

Gender, having a primary care doctor, having a family member who is a physician or who is a primary care doctor, and having already had a Family Medicine clerkship were not shown to impact student ratings.

**Table 6.** Effect of student age on perception of the importance of primary care.

Parameter	Estimate	Std. Error	Sig.	95%CI
Intercept	3.51	.027	.000	3.46–3.56
Post vs. Pre	.25	.032	.000	.19–.31
Age ≥26 years old	.073	.031	.021	.011–.13

## Discussion

In 2012, the Association of American Medical Colleges estimated that there would be a shortage of 66 000 primary care physicians by 2025 [17]. The Council on Graduate Medical Education has estimated a need for a workforce of at least 40% primary care physicians [14]. Medical schools have increased class sizes in response, but unless schools develop curricula to stimulate careers in primary care, that will do little to ease the shortage. Kernan et al. described The Selling of Primary Care 2015, and recommended eight medical school strategies to foster student interest in primary care, including (1) requiring clinical training in family medicine or primary care internal medicine, (2) placing students in high-performance primary care sites, (3) creating a school culture that values primary care, (4) creating strategic educational alliances with local practices, organizations, and groups that have or seek to develop high-performance primary care services, (5) supporting primary care teaching faculty, (6) putting student and resident education in primary care on an equal footing, (7) training students in systems-based analysis, including healthcare outcomes, to foster understanding of the implementation of effective primary care, and (8) training students in population-based healthcare [18].

We have seen that simply placing students in ambulatory offices and hoping for the best in terms of ‘selling’ primary care to students isn’t effective by itself. We believe that relying on busy primary care physicians to teach the principles of population-based health and quality care is shirking our responsibility as educators. We learned in the 1990s that when we gave feedback to students but didn’t call it ‘feedback’, students didn’t feel that they received feedback [19]. Providing a framework for effective naming and delivery of feedback changed that. Beverly et al. showed that providing intensive training in primary care to first year medical students can influence medical students’ attitudes toward primary care [20]. Our study looked at the impact of providing a curriculum during the Medicine clerkship to teach students the importance of primary care and then allowing them to experience in the clinic what they learned in the curriculum, providing a framework for students to be able to recognize the value and importance of primary care and helping to improve students’ attitudes toward primary care. In particular, highlighting the role of the primary care physician as patient advocate within the healthcare system can help students to understand the importance of primary care.

Students were asked on the survey, ‘Are you satisfied with your primary care physician?’, but were also asked this verbally as a group at the beginning of the intraclerkship. Those who were satisfied with their primary care physician reported verbally that they were satisfied with their access to care, felt that their physicians knew them well, and they were listened to when they had a problem. They also felt that their primary care physician helped them to navigate the health care system, and helped to coordinate their care. Those who responded affirmatively in writing that they were satisfied with their primary care physician were more likely to show an improvement in post-test attitudes toward primary care in the areas of primary care physicians improving the quality of care, making a difference in overall patient health, and finding primary care to be an intellectually challenging field. After being exposed to case scenarios in which primary care physicians negotiated differences of opinion with subspecialists to contribute to improved patient care, students were more likely to respond affirmatively that primary care internists need to stay involved in care provided to their patients by subspecialists, need to communicate medication changes to other physicians seeing the patient, and need to be willing to collaborate with specialists and hospitalists in patient care. The intraclerkship allowed students to be exposed to primary care physicians who practiced in varied practice settings, and who combined primary care with education, administration, or other areas of interest. After this exposure, students who were satisfied with the care provided by their own internists were more likely to recognize that primary care physicians can manage their workload and can shape the future of health care.

Older students were more likely to recognize the importance of communication between primary care physicians, specialists and hospitalists, and to view primary care as being as important as specialty care. One hypothesis is that older students may have had family and personal experiences in which primary care was pivotal in their own or their family’s health. Providing a forum for students to share personal experiences of the healthcare system may help to influence younger students’ attitudes toward primary care. Faculty may also be able to share their own experiences of the healthcare system, and to share how their own primary care physicians affected their own or their loved ones’ care.

In their 2008 study on factors associated with medical students’ career choices regarding internal medicine, Hauer et al. found that of students entering careers in Internal Medicine, only 19% responded that their Internal Medicine clerkship made a career in general internal medicine more attractive, while 48% responded that the clerkship made a career in subspecialty medicine more attractive [16]. By exposing students in the intraclerkship to excellent internists who are happy in their careers and who demonstrate the importance of work done by primary care internists, we believe our study shows that it is possible to provide an experience within the Medicine clerkship which positively influence attitudes toward primary care internal medicine.

Limitations of the study were that it didn’t measure whether the effects of the curriculum on attitudes toward primary care were long-lasting, or whether the curriculum ultimately affected career choice. Since the introduction of the curriculum in 2010, increasing numbers of students graduating from the University of Massachusetts Medical School have entered categorical Medicine or Medicine/Pediatrics residencies, but further study is needed to evaluate whether they ultimately choose careers in Primary Care Internal Medicine.

Meeting the demand of the future in healthcare is an important responsibility. Healthcare policymakers need to examine how to meet this need in the way we provide training, funding, and professional satisfaction to primary care physicians. Medical schools also have a responsibility to educate physicians who can fulfill the healthcare needs of our society. Providing innovative ways to communicate to students that a career in primary care provides great fulfillment, intellectual stimulation, and personal satisfaction can help to attract young physicians to this vitally important field.
